# Evaluating the 0–10 Point Pain Scale on Adolescent Opioid Use in US Emergency Departments

**DOI:** 10.3390/jcm11010038

**Published:** 2021-12-22

**Authors:** Michael T. Phan, Daniel M. Tomaszewski, Cody Arbuckle, Sun Yang, Brooke Jenkins, Michelle A. Fortier, Theodore Heyming, Erik Linstead, Candice Donaldson, Zeev Kain

**Affiliations:** 1 Department of Pharmacy Practice, Chapman University School of Pharmacy, Irvine, CA 92618, USA; syang@chapman.edu; 2 Department of Pharmaceutical and Health Economics, University of Southern California, Los Angeles, CA 90007, USA; dtomasze@healthpolicy.usc.edu; 3 Schmid College of Science and Technology, Chapman University, Orange, CA 92866, USA; arbuc100@gmail.com; 4 Department of Psychology, Crean College of Health and Behavioral Sciences, Chapman University, Orange, CA 92866, USA; bjenkins@chapman.edu (B.J.); Candice.donaldson@cgu.edu (C.D.); 5 Department of Anesthesiology and Perioperative Care, University of California Irvine, Irvine, CA 92697, USA; mfortier@hs.uci.edu (M.A.F.); zkain@hs.uci.edu (Z.K.); 6 UCI Center on Stress and Health, School of Medicine, University of California Irvine, Irvine, CA 92697, USA; 7 Sue and Bill Gross School of Nursing, University of California Irvine, Irvine, CA 92697, USA; 8 Department of Psychology, Children’s Hospital of Orange County, Orange, CA 92868, USA; 9 Department of Pediatric Emergency Medicine, Children’s Hospital of Orange County, Orange, CA 92868, USA; theyming@choc.org; 10 Fowler School of Engineering, Chapman University, Orange, CA 92866, USA; linstead@chapman.edu; 11 Department of Pediatrics, Children’s Hospital of Orange County, Orange, CA 92868, USA; 12 Yale Child Study Center, Yale University, New Haven, CT 06519, USA

**Keywords:** pain management, adolescent, emergency department, national, opioid, pain scale

## Abstract

Objective: To evaluate trends in national emergency department (ED) adolescent opioid use in relation to reported pain scores. Methods: A retrospective, cross-sectional analysis on National Hospital Ambulatory Medical Care Survey (NHAMCS) data was conducted on ED visits involving patients aged 11–21 from 2008–2017. Crude observational counts were extrapolated to weighted estimates matching total population counts. Multivariate models were used to evaluate the role of a pain score in the reported use of opioids. Anchors for pain scores were 0 (no pain) and 10 (worst pain imaginable). Results: 31,355 observations were captured, which were extrapolated by the NHAMCS to represent 162,515,943 visits nationwide. Overall, patients with a score of 10 were 1.35 times more likely to receive an opioid than patients scoring a 9, 41.7% (CI95 39.7–43.8%) and 31.0% (CI95 28.8–33.3%), respectively. Opioid use was significantly different between traditional pain score cutoffs of mild (1–3) and moderate pain (4–6), where scores of 4 were 1.76 times more likely to receive an opioid than scores of 3, 15.5% (CI95 13.7–17.3%) and 8.8% (CI95 7.1–10.6%), respectively. Scores of 7 were 1.33 times more likely to receive opioids than scores of 6, 24.7% (CI95 23.0–26.3%) and 18.5% (CI95 16.9–20.0%), respectively. Fractures had the highest likelihood of receiving an opioid, as 49.2% of adolescents with a fracture received an opioid (CI95 46.4–51.9%). Within this subgroup, only adolescents reporting a fracture pain score of 10 had significantly higher opioid use than adjacent pain scores, where fracture patients scoring a 10 were 1.4 times more likely to use opioids than those scoring 9, 82.2% (CI95 76.1–88.4%) and 59.8% (CI95 49.0–70.5%), respectively. Conclusions: While some guidelines in the adult population have revised cut-offs and groupings of the traditional tiers on a 0–10 point pain scale, the adolescent population may also require further examination to potentially warrant a similar adjustment.

## 1. Introduction

Acute pain is one of the primary reasons for visiting a US emergency department (ED) [1] and over 20% of adolescents visit a US ED each year [2]. When treating moderate to severe pain in this setting, healthcare providers may decide to use an opioid as treatment. While some opioid guidelines exist [3,4,5,6], healthcare providers must constantly weigh in the risks and benefits of using an opioid. These methods often rely on assessing factors such as diagnosis, age, treatment goals, and pain severity before prescribing an opioid [7]. One factor of interest is the use of patient-reported pain scores to assess pain severity and how that relates to the decision to prescribe an opioid.

Self-reported pain severity is a major factor used by clinicians to assess pain. Commonly used, these self-reporting tools are evidence of a patient’s experienced pain intensity [8]. However, the translation of these scores to treatment with analgesia are not as closely correlated as one would expect There is often a high degree of interpatient variability and reported pain can vary with factors such as age, sex, and ethnicity [9,10,11]. That said, current pain management guidelines unanimously agree that opioids should be reserved for moderate to severe pain, but it is still unclear how pain scores are being implemented in practice to guide key factors of prescribing opioids, such as duration of treatment, follow-up, evaluation of dose escalation, and discontinuation [3,5,6].

The development of opioid misuse and abuse may stem from the introduction of an opioid to individuals at an early age, which has prompted caution in providers caring for adolescent populations [12,13]. Up to 40% of adolescent emergency department patients are prescribed an opioid [14] and studies have shown variability in prescribing opioids within this setting [15,16,17,18]. Earlier studies using the Center for Disease Control and Prevention’s (CDC) National Hospital Ambulatory Medical Care Survey (NHAMCS) database have found that pediatric patients who reported higher pain severity had a higher likelihood of receiving an opioid analgesic over a non-narcotic analgesic [19,20]. In addition, pediatric patients without a documented pain score were just as likely to receive an opioid when mild pain was reported [20]. These studies used a four-tier pain severity scale: mild, moderate, severe, and no pain.

To investigate how a pain score may interact with the provider’s assessment of a patient, it is of interest to look at opioid prescribing rates in respect to a reported pain score. Therefore, our primary objective is to evaluate patterns between pain scores and the reported use of opioids to treat adolescents with pain within U.S. emergency departments. Our secondary objective is to further explore the landscape of opioid use with pain scores within subgroups such as diagnoses and age groups.

## 2. Methods

### 2.1. Study Design and Setting

This cross-sectional study analyzed data collected from the CDC’s NHAMCS. The latest available years of NHAMCS data, representing visits from 2008 to 2017, was downloaded and coded by the investigators in May 2020. Statistical analysis was done in June 2020. NHAMCS is conducted nationally in the US using a representative sample of visits to hospital-based outpatient clinics, emergency departments, and ambulatory surgery locations in non-institutional and short-stay hospitals. Field representatives collected data from medical records on patients’ symptoms, diagnoses, comorbidities, demographic characteristics, and medications ordered or provided. The survey employs a complex four-stage study design, designed to represent all ED visits nationwide. Crude observation counts were extrapolated to generate weighted estimates that matched the total population counts provided by the Census Bureau. A detailed description of the data collection methodology can be accessed at the CDC’s National Center for Health Statistics (NCHS) website [21]. The data in this deidentified, publicly available database did not constitute as human subjects research as defined by federal regulations and thereby exempted our study from IRB-approval.

### 2.2. Patient and Public Involvement

As this study is a secondary analysis on a dataset that was collected and published by the CDC, neither the patients nor the public were involved with providing input on the study design, conduct, dissemination, nor evaluation to produce this manuscript.

### 2.3. Selection of Population

All emergency department visits involving patients aged 11–21 years were included in the study and the study population was stratified into age groups of 11–14, 15–17, and 18–21 years old. This stratification was based on the American Academy of Pediatrics’ definitions of early, middle, and late adolescence [22].

Pain related visits were defined as visits with a documented pain diagnosis reported in the reasons for visit, reported as International Classification of Diseases, Ninth Revision, Clinical Modification codes. Pain diagnosis codes were established based on the clinical classification software categories available from the Agency for Healthcare Research and Quality Healthcare Cost and Utilization project. Visits that did not meet this criterion were identified as non pain related visits.

### 2.4. Variables

The primary outcome was dichotomous opioid use in the ED, reported as used or not used. Drugs were coded in terms of their generic component and therapeutic classification using Lexicon Plus^®^, North Kansas City, MO, USA, a comprehensive database of medications available in the U.S. market. The classification of opioids or non-opioids was determined using the CDC’s New Ambulatory Care Drug Database system [23].

Patient age, sex, race/ethnicity, insurance status (private, Medicaid, self-pay, other payment method) were recorded at each visit. Information on whether the episode was an initial visit or follow-up visit was also collected. Healthcare institution information was also collected to determine region (Midwest, Northeast, South, West), and whether it was in a metropolitan setting or not.

The NHAMCS uses a standard reason for visit classification to code complaints, symptoms, or other reasons for visit. The summary of codes and diagnoses can be found in the NHAMCS micro-data file documentation [24]. Included pain categories were abdominal pain, fractures, injury excluding fractures, and musculoskeletal pain. Musculoskeletal pain consisted of arthritis/joint pain, pelvic pain, back pain, and neck pain. Pain categories that did not meet the minimum of 30 visits in the unweighted sample were categorized under *other pain*. This group includes cancer-related pain, chest pain, cholelithiasis, headache, nephrolithiasis, sickle cell anemia, dental or jaw pain, fibromyalgia, and peripheral neuropathy.

### 2.5. Pain Score Measurement

Self-reported pain scores were assessed at triage using a numerical rating scale of 0–10. Anchors for the maximum and minimum scores were (0) no pain and (10) worst pain imaginable. Prior to 2008 the presenting level of pain was consolidated to no pain (numerical rating of 0), mild pain (numerical rating of 1–3), moderate pain (numerical rating of 4–6), and severe pain (numerical rating of 7–10) [24]. After 2008, stratified integer pain scores from 0–10 were reported per case. In this study, unknown pain scores were grouped together with scores of 0 due to the low counts of adolescent opioid patients and were excluded in the logistic regression model.

### 2.6. Statistical Analysis

All analyses were conducted on weighted data, as recommended by the CDC’s NCHS website. The weighting is calculated using the most recent census data to provide a stratified representation of the national patient population. All participants’ records were stored in a relational database using the open-source database software MySQL (v. 5.7.11, Oracle, Redwood Shores, CA, USA). All analytics were performed using the open-source statistical computing software R (v 3.2.3, R Foundation, Vienna, Austria). The functions of svydesign and svyglm from the R package survey were used to account for stratified, clustered, and weighted variables in the NHAMCS data. Wald tests of association were used to determine significance for bivariate analyses. Stepwise regression via backward elimination was used including all independent variables mentioned above. Separate independent logistic regression models were run holding pain score as the sole independent variable and opioid use as the dependent variable. CDC detailed documentation of the NHAMCS instrument, methodology and data files that were used as the basis for these analyses are available elsewhere [25].

## 3. Results

### 3.1. Participants

The overall sample for analysis (*n* = 31,355) represented a weighted estimate of 162,515,943 ED visits by adolescent patients (aged 11–21 years old) within the 10-year study period, from 2008 to 2017. Among the patients who received a pain score, 56% (*n* = 92,550,318) of the population was female and 44% (*n* = 69,965,625) were male. Approximately 53% (*n* = 89,125,196) were non-Hispanic White. Nearly half (*n* = 78,369,736) of the patients were late adolescents, aged 18–21, with 26% being (*n* = 41,730,020) mid-adolescents aged 15–17 and 26% (*n* = 42,416,187) being early adolescents aged 11–14. Within this patient population, there was a 31% reduction in opioid use between the two examined time intervals of 2008–2012 and 2013–2017 (Table 1).

### 3.2. Pain Scale Alone

**Figure 1:**
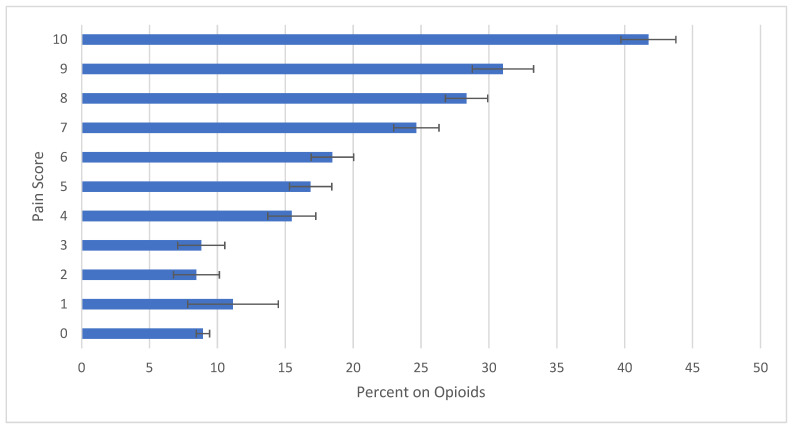
Proportion of Overall Opioid Prescribing per Pain Score Reported in US EDs from 2008 to 2017. Calculated using a 95% confidence interval.

Opioid rates were stratified into a more granular scale of 0 through 10 (Figure 1). The overall observed trend from the chart suggests that patients were more likely to be prescribed an opioid at higher pain scores. Scores of 0–3 did not statistically differ in opioid use rates. When a score of 4 was reached, patients were 1.76 times more likely to receive an opioid than a patient with a score of 3, 15.5% (CI95 13.7–17.3%) and 8.8% (CI95 7.1–10.6%), respectively. Among moderate pain scores, there was no difference in opioid prescribing rates, but again, when a score of 7 was reached (typically associated with a designation of severe pain), patients were 1.33 times more likely to receive an opioid than a patient with a score of 6, 24.7% (CI95 23.0–26.3%) and 18.5% (CI95 16.9–20.0%), respectively. Within the severe pain score range of 7–10, there were statistically significant increases in the likelihood of having opioid use reported. A score of 8 (28.3% (CI95 26.8–29.9%)) was 1.14 times more likely to receive an opioid than a 7. Although failing to reach statistical significance, a score of 9 (31.0% CI95 28.8–33.3%) was 1.1 times more likely to receive an opioid than a score of 8. A score of 10 (41.7% (CI95 39.7–43.8%)) was 1.35 times more likely to receive an opioid than a score of 9.

### 3.3. Diagnosis

**Figure 2:**
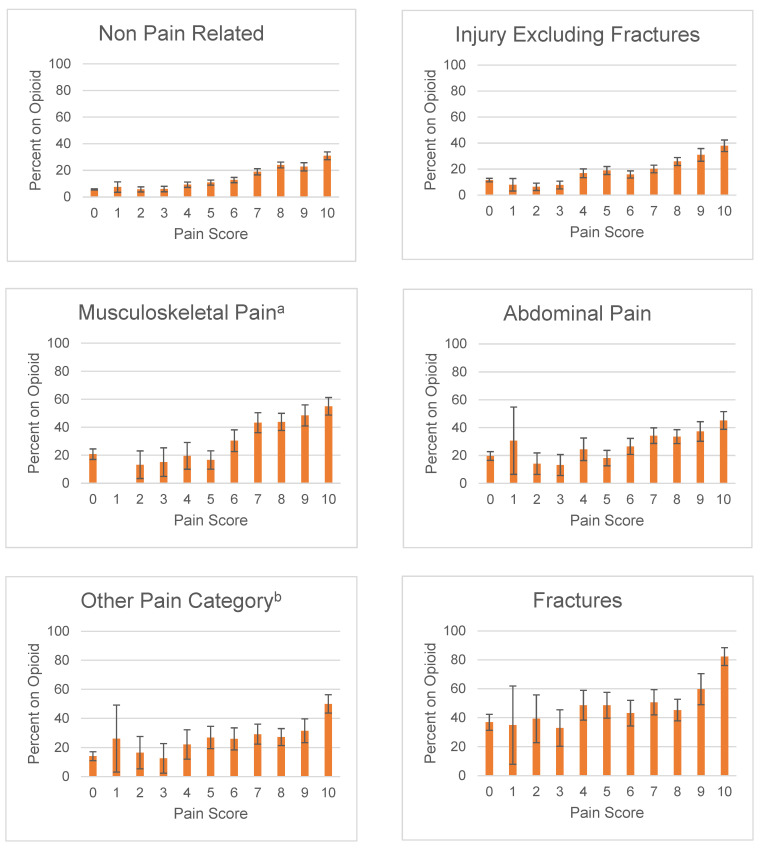
Proportion of Adolescent Opioid Use in the ED by Pain Score by Diagnosis. Calculated using a 95% confidence interval. ^a^ Musculoskeletal Pain includes arthritis/joint pain, pelvic pain, back pain, and neck pain. ^b^ Other Pain Category includes cancer-related pain, chest pain, cholelithiasis, headache, nephrolithiasis, sickle cell anemia, dental or jaw pain, fibromyalgia, and peripheral neuropathy.

In this study, more than half of the ED visits were reported as non-pain related. Of these visits, 11% (CI95 10.7–11.6%) received opioids. Of the visits that were pain related, 25% (CI95 24.5–25.9%) received opioids. Patients with fractures consistently received more opioids than other diagnoses with the same pain score. This second highest rate of prescription was for patients diagnosed with musculoskeletal pain (Figure 2). In this part of the analysis, pain scores of 0 and 1 within a diagnosis often did not meet the minimum threshold for significance due to the low number of adolescent patients who received an opioid at these scores.

#### 3.3.1. Fractures 

Pain related visits with fractures reported opioid use in 49% (CI95 46.4–51.9%) of visits. The likelihood of receiving an opioid appears to be unaffected by pain scores among patients who experienced a fracture and reported a pain score of 2–9, as none of the scores reached statistical significance from one another. A patient with a score of 10 (82.2% (CI95 76.1–88.4%)) was 1.4 times more likely to receive an opioid prescription than a patient with a score of 9 (59.8% (CI95 49.0–70.5%)).

#### 3.3.2. Musculoskeletal pain

In total, 34% (CI95 31.4–35.8%) of visits with musculoskeletal pain received an opioid. Visits with musculoskeletal pain that reported mild and moderate pain scores of 2–6 did not reach statistically significant differences among each other. Scores of 7 (43.3% (CI95 36.1–50.4%)) and higher were 2.6 times more likely to receive an opioid than scores of 5 (16.2% (CI95 10.1–23.2%)) or lower.

#### 3.3.3. Abdominal pain

Among visits with abdominal pain, 28% (CI95 25.7–29.3%) reported opioid use. For patients who reported pain scores of 7 and above, opioid use rates were similar across all severe pain scores and only when comparing to severe pain scores with mild or moderate pain scores was there a significant change in opioids use rate, where a pain score of 10 was 1.7 times more likely to receive an opioid than a score of 6, 45.2% (CI95 38.8–51.5%) and 26.6% (CI95 20.8–32.3%), respectively.

### 3.4. Patient Age

**Figure 3:**
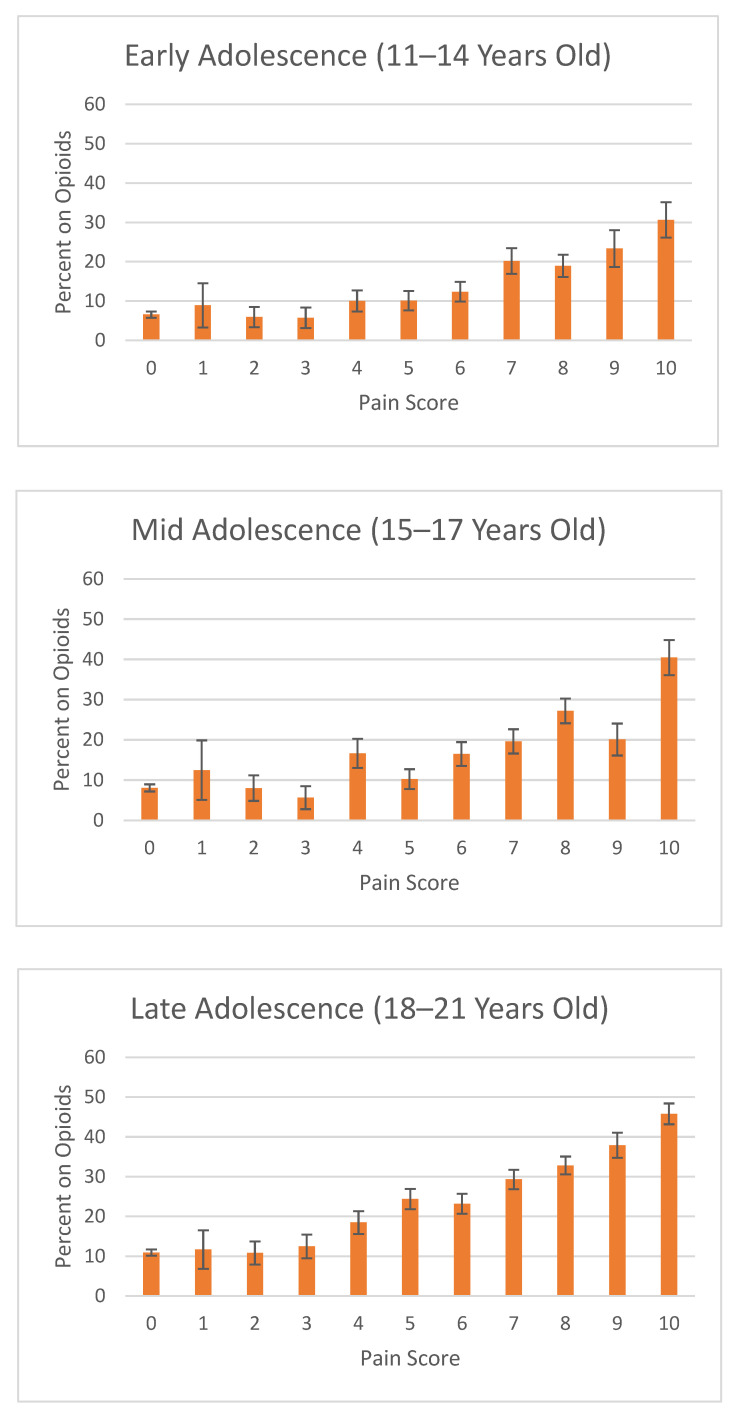
Proportion of Adolescent Opioid Use in the ED by Pain Score by Age Groups. Calculated using a 95% confidence interval. Age groups separated by early (11–14 years old), mid (15–17 years old), and late (18–21 years old) adolescents.

To examine the utility of pain scores when accounting for patient age, ED visits were subcategorized into three age groups (Figure 3). Within the late adolescent age group, the rate of receiving an opioid has an uptrend with an increasing pain score. Statistical significance was seen from score of 3, 4, and 5, 12.5% (CI95 9.5–15.5%), 18.5% (CI95 15.6–21.4%), and 24.4% (CI95 21.8–27.0%), respectively. A score of 10 (45.8% (CI95 43.2–48.4%)) was 1.2 times more likely to receive an opioid than late adolescents scoring a 9 (37.9% (CI95 34.7–41.0%)). Among the mid adolescent age group, the utility of the pain scores was less clear, with opioid use dropping at some of the higher reported pain scores. For instance, a pain score of 5 was 38% less likely to receive an opioid than a lower pain score of 4, 10.2% (CI95 7.8–12.7%) and 16.7% (CI95 13.1–20.3%), respectively. A pain score of 9 was less likely to receive an opioid than a pain score of 8, 20.1% (CI95 16.1–24.1%) and 27.2% (CI95 24.2–30.3%), respectively. Within the early adolescent patient population, there was a notable increase in opioid use at the moderate-severe pain score cutoff of 6 and 7, where early adolescent patients scoring a 7 (20.2% (CI9516.9–23.5%)) were 1.63 times more likely to receive an opioid than patients with a score of 6 (12.4% (9.9–14.9%)). A score of 10 (30.6% (CI95 26.1–35.1%)) was not found to be statistically different from a score of 9 (23.4% (CI95 18.7–28.1%)) but was 1.61 times more likely to receive an opioid than a score of 8 (19.0% (CI95 16.1–21.8%)). No differences were seen within moderate scores ranging from 4–6, nor within mild scores ranging from 1–3.

### 3.5. Patient Sex Differences

In male patients reporting a score of 4–10, representing moderate to severe pain, 57% (CI95 55–58%) were given an opioid, whereas 49% (CI95 48–50%) of female patients with a score of 4–10 were given an opioid. In severe pain scores of 8–10, higher proportions of male patients received opioids than females (35% (CI95 34–37%) vs. 31% (CI95 30–32%)).

### 3.6. Multivariate Analyses: Logistic Regression

A logistic regression analysis followed by backwards stepwise elimination resulted in a model of best fit that retained all loaded factors (Table 2). As expected, receiving a pain diagnosis increased the odds of receiving an opioid, with fractures and musculoskeletal pain having the highest odds of receiving an opioid, 6.65 (CI95 5.29–8.36) and 2.57 (CI95 2.10–3.14), respectively. Pain scores were also shown to be statistically significant, with patients being 1.25 times more likely to receive an opioid with each additional point on a pain scale (CI95 1.22–1.28). Male patients were 19% more likely to receive an opioid (OR 0.81 CI95 0.72–0.92). Age also contributed largely to the model, where older age groups showed statistically significant odds ratios. Race and ethnicity did not reach statistical significance in this model.

## 4. Discussion

To our knowledge, our study is the first to utilize the 0–10 numeric rating scale data in the NHAMCS dataset to examine opioid prescribing among adolescent emergency department (ED) visits. Our data suggests that even with a 10 point scale, prescribers may apply the traditional four-tier model for pain assessment, with the exception of a pain score of 10, which is distinguished from the other pain scores from the “severe” tier. Reported rates of opioid use versus independent pain scores showed a significant increase in opioid use rates from 3 to 4 and 6 to 7, which is consistent with earlier NHAMCS studies that examined pain intensity as mild, moderate, and severe pain. This trend may be due to the general perception in clinical practice that these scores represent cutoffs between mild, moderate, and severe pain. However, defining pain severity using a 0–10 pain scale has been poorly studied. To our knowledge, the generally accepted current cutoffs, 0 (none); 1–3 (mild); 4–6 (moderate); and 7–10 (severe), were defined with the assumption that it would be universally understood by both patient and provider [5]. Yet, studies have demonstrated that there is a high degree of varying interpatient interpretability of pain scales, resulting in different conclusions regarding the defined cutoffs for mild, moderate, and severe pain [26,27,28]. While a 10-point scale may provide more flexibility and insight on the degree of pain a patient feels, the interpretation of the score should be used cautiously and within a holistic context of the patient’s experience of pain.

Our results showed that there was a statistically significant difference in opioid prescribing for patients who scored a 10 compared to other “severe” scores, i.e., 7–9. This was also observed within our subgroup analyses, where a score of 10 would regularly be statistically different from other severe pain scores. These results challenge the interpretation that a score of 10 should be categorized in the same group as a score of 7–9. Likewise, there were a few occurrences where statistically different opioid rates existed within the moderate pain scores of 4–6. Though not statistically significant, there appeared to be a higher likelihood of opioid use for a pain score of 1 than a score of 2 or 3. The trend could be driven by other important factors such as diagnosis. Scores of 2 or 3 could consist more so of visits associated with diagnoses that are better managed with non-narcotics. For example, acute otitis media is better managed with an NSAID than an opioid. Although it is unclear whether prescribers unintentionally utilize a different pain scale categorization from the traditional 4-tier model, the data suggests that prescribers practice as though there is a 5th tier that separates a score of 10, the worst pain imaginable, from other severe pain scores. The way prescribers utilize lower tier pain scores to determine overall analgesic use is not well defined in this study, as few patients used opioids for lower pain scores. Adjustments to the categorization of these pain score ranges is a continual process; in recent years, the NHAMCS revised their guidelines to redefine pain score interpretation, further separating the scores into a six tier system; no pain (0–1), mild pain (2–3), discomforting moderate pain (4–5), distressing-severe pain (6–7), intense-very severe pain (8–9), and unbearable pain (10) [29]. Even though the adoption of these pain score interpretations is yet to be widespread with regard to documentation, our study would suggest that emergency department providers already practice as if there was a different scale system in use.

The role of a pain score within the overarching umbrella of pain assessment is nuanced and sometimes does not predict the delivery of analgesia. While it could be expected that there is a general positive relationship between higher pain score and opioid use or prescribing, studies have suggested that the correlation may sometimes be weak [30,31,32]. In one study examining emergency department visits of patients aged 3–20, there was no statistical difference in analgesia administered nor time to administer the analgesia in patients who reported a pain score of 6 or higher [31]. Our study had similar results when opioid use rates were subcategorized by diagnosis. We found that within certain diagnoses, opioid-use rates did not change over the span of pain scores that ranged from mild to severe pain. Patients with fractures were more likely to receive opioids compared to other diagnoses, but among patients with fractures, only patients scoring a 10 were more likely to receive an opioid while scores ranging from 2–9 did not reach any statistical difference in opioid rates. Rates with musculoskeletal pain increased between moderate pain and severe pain, but there was no statistical difference once they reported a score of 7 or higher.

The output of the logistic regression model suggests that clinical factors that largely contribute to the likelihood of receiving an opioid were diagnosis, sex, and age. Severe conditions such as fractures and musculoskeletal pain had higher odds of receiving an opioid. Sex-based differences were also observed in our subgroup analysis, showing that males were likely to receive an opioid than females with similar pain scores. The underlying cause of this disparity is indeterminate. However, differences in pain threshold between genders, as well as provider perceptions on how genders report pain differently, may be driving factors. The idea of age as a factor in pain management was supported with our subgroup analysis, demonstrating an increase in opioid use among late adolescent patients across pain scores. Treatment for the late adolescent group can be addressed using some guidelines intended for the adult population, which may be a reason to why the late adolescents had a steady trend with pain score. Meanwhile, the trend was less clear with the younger age groups as there were statistically significant drops in opioid use rates with some higher pain scores. The juxtaposition of different trends suggests that the reported pain scores of older patients may generally be viewed as more reliable than younger patients’ reports, thereby suggesting that opioid prescription rates correlate with pain scores better among older ages than younger ages. However, other considerations may contribute to this trend, such as provider discomfort with opioid dosing in younger patients due to a lack of guidelines with this vulnerable population and the potential increased risk associated with opioid use in younger patients.

These findings were similar to other reports. Two other studies examining pediatric ED opioid used identified age as a factor; both studies demonstrated decreased opioid use in younger patients [33,34]. One study examining opioid use in North American EDs–Canada and the United States demonstrated that more than 60% of providers regularly used codeine-containing products for severe musculoskeletal pain in patients aged 15 years old, and more than 30% used oxycodone-containing products for the same severe pain scenario [34]. The rates of provider preference regarding opioid use is consistent with our finding that patients reporting severe musculoskeletal pain are likely to receive opioid therapy. However, using opioids for musculoskeletal pain in this population is a debated method and current guidelines require additional research to improve the strategy [35].

Our results showed that certain pain scores within a range had similar opioid prescribing rates, suggesting that prescribers are less concerned with the precise score reported and instead are more focused on categorizing the degree of severity of patient pain. The traditional 4-tier model of defining pain severity–mild, moderate, severe, and no pain–does not fit well with our results, as a score of 10 receives significantly more opioids than scores of 7–9, which typically indicate severe pain. Future prospective studies are warranted to identify how a traditional 4-tier model, the NHAMCS 6-tier model, and other models hold up to analysis within a 0–10 point scale. Finding more accurate pain severity categories may improve triage and screening practices and may be more accurate for reflecting the actions that clinicians must intuitively perform with a 0–10 point scale score.

Additionally, defining pain score groupings may not only benefit prescribing decisions but also help highlight prescribing discrepancies. Our data showed that some patients received opioids despite reporting mild pain, which is an opportunity for institutions to implement procedures of review to ensure appropriate opioid use. Likewise, a similar systematic review process may be used for instances where patients reporting a score of 10 receive a non-opioid analgesic instead of an opioid, ensuring that the decision is appropriate.

## 5. Limitations

The NHAMCS database only captures the presenting pain score, which therefore limits the inference between pain score severity and opioid use. Information on the way in which the opioid was used is also a limitation in this database, as it was unclear if opioids were used empirically or for rescue therapy. The strength, frequency, dose, and timing of opioid use are also missing. Opioids were evaluated as a homogenous group instead of independent product of active ingredients due to the low cohort sizes. While information on non-opioid analgesics is available within this dataset, it is unclear as to whether they were used adjunctly or separately from the opioids. Other important considerations that may be missed in our dataset include factors such as social history of drug misuse, location of injury, presence of mood disorder, and allergies to either non-opioid or opioid analgesics. In addition, other external factors may exist that influence provider decision making, such as the type of hospital–academic versus community, pediatric versus adult—as well as, health system protocols that provide guidelines for opioid use, as well as inter-provider variability, where some providers may more or less be comfortable with utilizing narcotics in this population. Furthermore, the pain score was collected at triage, and it is unclear whether providers were required to know the patient’s pain score while making their clinical assessment.

## 6. Conclusions

Our examination of incremental pain scores suggests that, among the pain scores associated with severe pain, a score of 10 significantly receives more opioids than the rest of the severe pain scores of 7–9 reported from adolescents, whereas lower scores have maintained consistent rates of opioid use within the respective groupings of mild and moderate pain. While some guidelines have made this adjustment for the adult setting and have separated a 10, the worst pain imaginable, from other severe pain scores, its application to adolescent patients is yet to be fully understood. Furthermore, our subgroup analysis suggested that the pain score relationship with opioid use was more nuanced and that other clinical factors, such as diagnosis and patient age, affect the application of a pain score for opioid use in the ED. Future research should examine additional factors that may contribute to the prescriber decision-making process of using an opioid in this population and setting.

## Figures and Tables

**Table 1:** Demographic Characteristics of a Weighted Sample of Adolescent Emergency Department Visits in the US from 2008 to 2017 ^a^.

Variable	Total Patients	Percent on Opioid (CI95)
Sex		
Female	92,550,318	16.8% (17.3–16.2%)
Male	69,965,625	18.1% (18.8–17.5%)
Race/Ethnicity		
Black	39,255,551	14.1% (14.8–13.3%)
Hispanic	29,956,070	15.7% (16.6–14.7%)
Other	4,179,126	18.3% (20.7–15.9%)
White	89,125,196	19.3% (19.9–18.7%)
Age		
11–14	42,416,187	11.3% (12.0–10.7%)
15–17	41,730,020	14.7% (15.5–13.9%)
18–21	78,369,736	22% (22.7–21.4%)
MSA		
Metropolitan area	120,279,731	17.9% (18.4–17.4%)
Non-metropolitan area	24,884,524	15.5% (16.6–14.4%)
Unknown	17,351,688	16.5% (17.6–15.3%)
Episode Of Care		
Initial visit to this ED	144,282,632	17.3% (17.7–16.8%)
Follow-up visit to this ED	7,330,229	20.9% (22.9–18.9%)
Unknown/Missing Data	10,903,082	15.9% (17.4–14.4%)
Time Interval		
2008–2012	73,585,030	20.9% (21.5–20.3%)
2013–2017	88,930,913	14.4% (15.0–13.8%)
Prolonged Visit		
Not Prolonged	109,946,374	18.9% (19.4–18.4%)
Prolonged Visit	8,829,988	25.6% (27.5–23.8%)
Unknown/Missing Data	43,739,581	11.8% (12.6–11.0%)

^a^ Calculated using 95% confidence intervals; MSA, Metropolitan Statistical Area; ED, Emergency Department.

**Table 2:** Logistic Regression Analysis of Predictors of Adolescent ED Opioid Use.

		Odds Ratio (CI95)
	(Intercept)	0.014 (0.009–0.023)
Ethnicity	Other	
	Black	0.775 (0.550–1.093)
	Hispanic	0.992 (0.713–1.379)
	White	1.241 (0.904–1.703)
Diagnosis	Non Pain Related	
	Abdominal Pain	1.98 (1.650–2.375) *
	Fractures	6.653 (5.289–8.369) *
	Injury excluding fracture	1.391 (1.211–1.598) *
	Musculoskeletal Pain	2.573 (2.105–3.144) *
	Other Pain Category	1.845 (1.498–2.271) *
Sex	Male	
	Female	0.811 (0.716–0.918)
Payment Method	Private Insurance	
	Medicaid, CHIP, State	0.760 (0.668–0.865)
	Other Payment Method	0.832 (0.685–1.01)
	Self-Pay	1.058 (0.895–1.25)
Adolescent Age	11–14	
	15–17	1.424 (1.203–1.687) *
	18–21	2.399 (2.059–2.795) *
Prolonged ED visit	Not Prolonged	
	Prolonged	1.700 (1.405–2.057) *
	Missing Data	0.647 (0.552–0.759)
Region	Northeast	
	Midwest	1.674 (1.353–2.072) *
	South	2.076 (1.723–2.501) *
	West	2.613 (2.089–3.267) *
Pain Scale	PAINSCALE	1.253 (1.224–1.283) *
Episode of Care	Initial visit to this ED	
	Follow-up visit to this ED	1.408 (1.120–1.771) *
	Unknown/Missing Data	1.094 (0.807–1.485)
Metropolitan Area	Non Metropolitan Area	
	Metropolitan Area	1.237 (1.003–1.526) *
	Unknown	0.936 (0.72–1.217)

* denotes significance using a 95% confidence interval.

## Data Availability

The National Hospital Ambulatory Medical Care Survey can be freely obtained at the following website https://www.cdc.gov/nchs/ahcd/datasets_documentation_related.htm accessed on 1 May 2020.
